# The Geometry of Marked Contact Engel Structures

**DOI:** 10.1007/s12220-020-00545-5

**Published:** 2020-11-19

**Authors:** Gianni Manno, Paweł Nurowski, Katja Sagerschnig

**Affiliations:** 1 Politecnico di Torino, Turin, Italy; 2 Center for Theoretical Physics PAS, Warsaw, Poland

**Keywords:** Special contact structures, Foliations, $G_2$, Double fibration, Cartan’s equivalence method, Local invariants, Tanaka prolongation

## Abstract

A *contact twisted cubic structure* $({\mathcal M},\mathcal {C},{\varvec{\upgamma }})$ is a 5-dimensional manifold ${\mathcal M}$ together with a contact distribution $\mathcal {C}$ and a bundle of twisted cubics ${\varvec{\upgamma }}\subset \mathbb {P}(\mathcal {C})$ compatible with the conformal symplectic form on $\mathcal {C}$. The simplest contact twisted cubic structure is referred to as the *contact Engel structure*; its symmetry group is the exceptional group $\mathrm {G}_2$. In the present paper we equip the contact Engel structure with a smooth section $\sigma : {\mathcal M}\rightarrow {\varvec{\upgamma }}$, which “marks” a point in each fibre ${\varvec{\upgamma }}_x$. We study the local geometry of the resulting structures $({\mathcal M},\mathcal {C},{\varvec{\upgamma }}, \sigma )$, which we call *marked contact Engel structures*. Equivalently, our study can be viewed as a study of foliations of ${\mathcal M}$ by curves whose tangent directions are everywhere contained in ${\varvec{\upgamma }}$. We provide a complete set of local invariants of marked contact Engel structures, we classify all homogeneous models with symmetry groups of dimension $\ge 6$ up to local equivalence, and we prove an analogue of the classical Kerr theorem from Relativity.

## The $G_2$-Geometries of Cartan and Engel

In 1893 Cartan and Engel, in the same journal but independent articles [4, 7], provided explicit realizations of the Lie algebra of the exceptional Lie group $\mathrm {G}_2$ as infinitesimal automorphisms of differential geometric structures on 5-dimensional manifolds. (In this paper $\mathrm {G}_2$ denotes a Lie group whose Lie algebra is the split real form of the complex exceptional simple Lie algebra $\mathfrak {g}_2$.) One of these structures was the simplest (2, 3, 5) *distribution*, that is, rank 2 distribution $\mathfrak {D}\subset T\mathcal {N}^5$ on a 5-manifold $\mathcal {N}^5$ such that $[\mathfrak {D},\mathfrak {D}]$ is a rank 3 distribution and $[\mathfrak {D},[\mathfrak {D},\mathfrak {D}]]=T\mathcal {N}^5$. These non-integrable distributions form an interesting and well studied (local) geometry, see Cartan’s classical paper [5] and e.g. [11] for more recent work and the associated conformal geometry.

The other structure was the simplest contact twisted cubic structure. Consider a smooth 5-dimensional manifold ${\mathcal M}^5$ together with a contact distribution, i.e., a rank 4 subbundle $\mathcal {C}\subset T{\mathcal M}^5$ such that the Levi bracket

1.1 $$\begin{aligned} \mathcal {L}:\Lambda ^2\mathcal {C}\rightarrow T{\mathcal M}^5/\mathcal {C},\quad \xi _x\wedge \eta _x\mapsto [\xi ,\eta ]_x \mathrm {mod} \mathcal {C}_x \end{aligned}$$

is non-degenerate at each point $x\in {\mathcal M}^5$. Then $\mathcal {L}_x$ endows each fibre $\mathcal {C}_x$ with the structure of a conformal symplectic vector space. Consider further a sub-bundle ${\varvec{\upgamma }}\subset \mathbb {P}(\mathcal {C})$ in the projectivization of $\mathcal {C}$ such that each fibre ${\varvec{\upgamma }}_x\subset \mathbb {P}(\mathcal {C}_x)$ is the image of a map

$$\begin{aligned} \mathbb {R}\mathbb {P}^1\rightarrow \mathbb {P}(\mathcal {C}_x)\cong \mathbb {R}\mathbb {P}^3,\quad [t,s]\mapsto [t^3,t^2 s,t s^2,s^3]\,; \end{aligned}$$

such a curve ${\varvec{\upgamma }}_x$ is called a twisted cubic curve (or rational normal curve of degree three). Assume that the twisted cubic is Legendrian, which means that it is compatible with the conformal symplectic structure on the contact plane (see Sect. 2.2 for details). Then $({\mathcal M}^5,\mathcal {C},{\varvec{\upgamma }})$ is called a *contact twisted cubic structure*.

Both geometries, (2, 3, 5) distributions as well as contact twisted cubic structures, are examples of *parabolic geometries*, see [6]. As such, they admit canonical Cartan connections, whose curvature gives rise to the fundamental invariants of these structures. If the curvature of a given structure identically vanishes, then the structure is locally equivalent to the *flat model* of the geometry under consideration: In case of a (2, 3, 5) distribution this is the $\mathrm {G}_2$-invariant (2, 3, 5) distribution on the flag manifold $\mathrm {G}_2/\mathrm {P}_1$ and in case of a contact twisted cubic structure this is the $\mathrm {G}_2$-invariant contact twisted cubic structure on the flag manifold $\mathrm {G}_2/P_2$. Here we use the standard notation $\mathrm {P}_1$ and $\mathrm {P}_2$ for the two 9-dimensional maximal parabolic subgroups of $\mathrm {G}_2$. The geometric structures presented by Cartan and Engel are local coordinate description of the two flat models.

Engel’s description of the $\mathrm {G}_2$-invariant contact twisted cubic structure was (up to a different choice of coordinates) as follows: Let $(x^0,x^1,x^2,x^3,x^4)$ be local coordinates $\mathcal {U}\subset \mathbb {R}^5$ and consider the coframe

1.2 $$\begin{aligned} \alpha ^0=\mathrm {d}x^0+x^1 \mathrm {d}x^4- 3 x^2 \mathrm {d}x^3,&\quad \alpha ^1=\mathrm{d}x^1,\quad \alpha ^2=\mathrm{d}x^2,\nonumber \\&\quad \alpha ^3=\mathrm{d}x^3,\quad \alpha ^4=\mathrm{d}x^4, \end{aligned}$$

with dual frame

1.3 $$\begin{aligned} X_0=\partial _{x^0},\quad X_1=\partial _{x^1}, \quad X_2=\partial _{x^2},&\quad X_3=3x^2\partial _{x^0}+\partial _{x^3},\nonumber \\&\quad X_4=-x^1\partial _{x^0}+\partial _{x^4}. \end{aligned}$$

Here $\alpha ^0$ is a contact form and defines a contact distribution $\mathcal {C}=\mathrm {ker}(\alpha ^0)$. Now consider the set of horizontal null vectors

$$\begin{aligned} {\hat{\varvec{\upgamma }}}=\{\,Y\in \mathcal {C}:\,g_1(Y,Y)=g_2(Y,Y)=g_3(Y,Y)=0\,\} \end{aligned}$$

of the three degenerate metrics

1.4 $$\begin{aligned} g_1=\alpha ^1\alpha ^3- (\alpha ^2)^2,\quad g_2= \alpha ^2\alpha ^4- (\alpha ^3)^2,\quad g_3= \alpha ^2\alpha ^3- \alpha ^1 \alpha ^4, \end{aligned}$$

where $\alpha ^i\alpha ^j=\tfrac{1}{2}(\alpha ^i\otimes \alpha ^j+\alpha ^j\otimes \alpha ^i)$. Then $Y\in \Gamma (\mathcal {C})$ takes values in ${\hat{\varvec{\upgamma }}}$ if and only if is of the form

$$\begin{aligned} Y=t^3 X_1+t^2 s X_2+ ts^2X_3+s^3X_4. \end{aligned}$$

Hence the projectivization ${\varvec{\upgamma }}_x\subset \mathbb {P}(\mathcal {C}_x)$ of ${\hat{\varvec{\upgamma }}}_x$ is a twisted cubic curve, and it is straightforward to verify that it is Legendrian. A contact twisted cubic structure that is locally equivalent to the $\mathrm {G}_2$-invariant structure $(\mathcal {U},\mathcal {C},{\varvec{\upgamma }})$ described above will be called a *contact Engel structure*.^1^

## Marked Contact Engel Structures and a Kerr Theorem

On a contact Engel structure there is, at each point $x\in \mathcal {M}^5$, a distinguished set of directions, namely those corresponding to points $p\in {\varvec{\upgamma }}_x$. In this work, we equip the contact Engel structure (possibly after restricting to an open subset of $\mathcal {M}^5$) with a section $\sigma$ that marks a point $*=\sigma (x)$ in each twisted cubic ${\varvec{\upgamma }}_x$.

### Definition 1

A *marked contact Engel structure* $(\mathcal {U},\mathcal {C},{\varvec{\upgamma }},\sigma )$ is a contact Engel structure together with a smooth section

$$\begin{aligned} \sigma :\mathcal {U}\rightarrow {\varvec{\upgamma }}\subset \mathbb {P}(\mathcal {C}) \end{aligned}$$

of the bundle $\,\mathbb {R}\mathbb {P}^1\rightarrow {\varvec{\upgamma }}\rightarrow \mathcal {U}$ of twisted cubics.

Since ${\varvec{\upgamma }}_x\subset \mathbb {P}(\mathcal {C}_x)$ is cut out by the three polynomials (1.4) and because of the analogy with Lorentzian geometry to be discussed below, we refer to directions in ${\varvec{\upgamma }}$ as *null directions*.^2^ A marked contact Engel structure can be thought of as a *null congruence structure*, that is, a (local) foliation of the contact Engel structure by horizontal null curves. For each $x\in \mathcal {U}$, the point $\sigma (x)\in {\varvec{\upgamma }}_x$ corresponds to a null direction $\ell ^{\sigma }_x$ in the contact plane $\mathcal {C}_x$. Therefore the section $\sigma$ defines a rank one distribution $\ell ^{\sigma }\subset T\mathcal {U}$ whose integral curves define the null congruence.

### Analogy with Null Congruence Structures in Lorentzian Geometry

Conformal Lorentzian geometries $({\mathcal M}^4,[g])$ in 4-dimensions are the geometries studied in General Relativity when the related physics is concerned with massless particles only. Conformal geometries are also examples of parabolic geometries, just like contact twisted cubic structures, which are the background geometries of this paper.

Of particular importance in General Relativity are *null congruences*, i.e. *foliations* of $({\mathcal M}^4,[g])$ *by null curves*. A conformal Lorentzian manifold equipped with a null congruence is called a *null congruence structure*. We want to point out here that many well-known results from General Relativity that are concerned with null congruences (such as the Kerr theorem and the Goldberg-Sachs theorem, see e.g. [12, 14, 15, 17]) have interesting analogies in the general framework of parabolic geometries. For the reader familiar with the results from Relativity, we note that in the case considered here the analogy is as follows:

**Table:** 

Conformal spacetime	Contact twisted cubic structure
Conformally flat spacetime	Engel structure
Conformally flat null congruence structure	Marked contact Engel structure
Conformally flat null congruence structure of geodesics	Integrable marked contact Engel structure (see Definition 2)
Conformally flat null congruence structure of shearfree geodesics	Integrable marked contact Engel structure
Robinson congruence	Maximal and submaximal models (see Sect. 2.7)

Before introducing the central notion of an integrable marked contact Engel structure, we summarize the following algebraic preliminaries about Legendrian twisted cubics.

### Algebraic Preliminaries

The *twisted cubic* $\upgamma \subset \mathbb {R}\mathbb {P}^3$ is the image of the Veronese map

2.1 $$\begin{aligned} \textstyle {\mathbb {R}\mathbb {P}^1=\mathbb {P}(\mathbb {R}^2)\rightarrow \mathbb {P} (\smash {\bigodot ^3\mathbb {R}^2})=\mathbb {R}\mathbb {P}^3,\quad [w]\mapsto [w\odot w\odot w].} \end{aligned}$$

In coordinates with respect to bases $(e_1,e_2)$ of $\mathbb {R}^2$ and $(E_1,E_2,E_3,E_4)$ of $\smash {\bigodot ^3\mathbb {R}^2}$, where $E=e_1\odot e_1\odot e_1, E_2=3 e_1\odot e_1\odot e_2, E_3=3 e_1\odot e_2\odot e_2, E_4=e_2\odot e_2\odot e_2,$ it can be parameterized as $[s,t]\mapsto [s^3,s^2 t,s t^2,t^3].$ Alternatively, denoting by $(E^1, E^2, E^3, E^4)$ the dual basis, the twisted cubic is given by the zero locus of

2.2 $$\begin{aligned} g_1=E^1E^3-(E^2)^2,\quad g_2=E^2 E^4-(E^3)^2, \quad g_3=E^2E^3- E^1E^4. \end{aligned}$$

With respect to the introduced bases, the irreducible representation

2.3 $$\begin{aligned} \textstyle {\phi :\mathrm {GL}(2,\mathbb {R})\rightarrow \mathrm {GL}(4,\mathbb {R}) =\mathrm {Aut}(\smash {\bigodot ^3\mathbb {R}^2}),} \end{aligned}$$

of $\mathrm {GL}(2,\mathbb {R})$ is of the form

2.4 $$\begin{aligned} \begin{pmatrix}\alpha &{}\beta \\ \rho &{}\delta \end{pmatrix}\mapsto \begin{pmatrix}\alpha ^3&{}3\alpha ^2\beta &{}3\alpha \beta ^2&{}\beta ^3\\ \alpha ^2\rho &{}\alpha ^2\delta +2\alpha \beta \rho &{}2\alpha \beta \delta +\beta ^2\rho &{}\beta ^2\delta \\ \alpha \rho ^2&{}2\alpha \delta \rho +\beta \rho ^2&{}\alpha \delta ^2+2\beta \delta \rho &{}\beta \delta ^2\\ \rho ^3&{}3\delta \rho ^2&{}3\delta ^2\rho &{}\delta ^3\end{pmatrix}. \end{aligned}$$

The $\mathrm {GL}(2,\mathbb {R})$-decomposition $\bigwedge ^2(\smash {\bigodot ^3\mathbb {R}^2})\cong \bigodot ^4\mathbb {R}^2\oplus \mathbb {R}$ shows that there is a unique (up to scalars) skew-symmetric bilinear form on $\mathbb {R}^4=\smash {\bigodot ^3\mathbb {R}^2}$ preserved by the $\mathrm {GL}(2,\mathbb {R})$-action up to scalars. It is given by

2.5 $$\begin{aligned} \omega =E^1\wedge E^4 - 3 E^2\wedge E^3.\end{aligned}$$

In order to characterize the $\mathrm {GL}(2,\mathbb {R})$-invariant conformal class of the symplectic form (2.5) in terms of the twisted cubic, we shall introduce some more terminology: Let $\omega$ be a symplectic form on $\mathbb {R}^4$ and let $[\omega ]$ be the conformal class of all non-zero multiples of $\omega$. Recall that a maximal subspace $\mathbb {W}$ on which a symplectic form $\omega$ (and then any $\omega '\in [\omega ]$) vanishes identically is called *Lagrangian*. A twisted cubic $\upgamma \subset \mathbb {P}(\mathbb {R}^4)$ is called *Legendrian* with respect to $[\omega ]$, see [3], if the cone

$$\begin{aligned} \hat{\upgamma }=\{\,w\odot w\odot w : w\in \mathbb {R}^2\,\}\subset \mathbb {R}^4 \end{aligned}$$

is Lagrangian, i.e., the tangent space at each point $\hat{p}$ of $\hat{\upgamma }\setminus \{0\}$ is a Lagrangian subspace of $T_{\hat{p}}\mathbb {R}^4\cong \mathbb {R}^4$. The conformal symplectic structure $[\omega ]$ generated by $\omega =E^1\wedge E^4 - 3 E^2\wedge E^3$ is the unique conformal symplectic structure such that $\upgamma =[s^3,s^2 t,s t^2,t^3]$ is Legendrian with respect to $[\omega ]$.

### The Contact Engel Structure in the Root Diagram

A reader familiar with reading root diagrams, can see the $\mathrm {G}_2$-invariant contact Engel structure on $\mathrm {G}_2/P_2$ in the root diagram for $\mathrm {G}_2$.


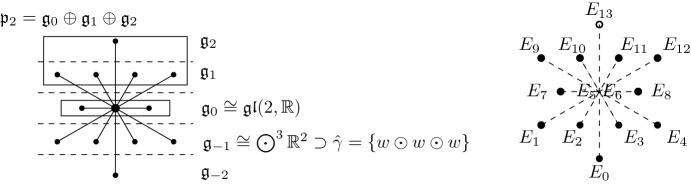


On the right, the $E_i$ denote root vectors in the corresponding root spaces. The parabolic subalgebra $\mathfrak {p}_2\subset \mathfrak {g}$ is spanned by the root vectors $E_5, \dots ,E_{13}.$ The tangent space of $\mathrm {G}_2/P_2$ at the identity can be identified with $\mathfrak {g}/\mathfrak {p}_2$. As a representation of the Levi factor $\mathrm {G}_0\cong \mathrm {GL}(2,\mathbb {R})$ of $P_2$, we have $\mathfrak {g}/\mathfrak {p}_2\cong \mathfrak {g}_{-2}\oplus \mathfrak {g}_{-1}$. Here $\mathfrak {g}_{-1}=\mathrm {span}(E_1,E_2,E_3,E_4)$ is an irreducible subrepresentation, which can be identified with $\smash {\bigodot ^3\mathbb {R}^2}$. It corresponds to the $\mathrm {G}_2$-invariant contact distribution $\mathcal {C}\subset T(\mathrm {G}_2/P_2)$. The bundle of twisted cubics ${\varvec{\upgamma }}\subset \mathbb {P}(\mathcal {C})$ then corresponds to the highest weight orbit in $\mathbb {P}(\mathfrak {g}_{-1})$ consisting of lines through simple vectors in $\smash {\bigodot ^3\mathbb {R}^2}$.

### The Osculating Filtration and Integrability

Now suppose the twisted cubic is marked, that is, a point $p\in \upgamma \subset \mathbb {P}(\mathbb {R}^4)$ is distinguished. The point *p* corresponds to a line $\ell \subset \hat{\upgamma }\subset \mathbb {R}^4\cong \smash {\bigodot ^3\mathbb {R}^2}$, which is of the form $\ell =\mathrm {Span}(\{l\odot l\odot l :l\in L\})$ for a unique 1-dimensional subspace $L \subset \mathbb {R}^2$. It further determines a 2-dimensional subspace $\mathrm {D}=\mathrm {Span}(\{l\odot l\odot e$ : $l\in L, \,e\in \mathbb {R}^2\})$, and a 3-dimensional subspace $\mathrm {H}=\mathrm {Span}(\{l\odot e\odot f$ : $l\in L, \,e,f\in \mathbb {R}^2\})$ of $\mathbb {R}^4=\smash {\bigodot ^3\mathbb {R}^2}$. Geometrically, $\mathrm {D}$ is the de-projectivized tangent line to $\upgamma$ at *p* and $\mathrm {H}$ is the de-projectivized osculating plane to $\upgamma$ at *p*. Thus we refer to the filtration

2.6 $$\begin{aligned} \ell \subset \mathrm {D}\subset \mathrm {H}\subset \mathbb {R}^4. \end{aligned}$$

as the *osculating filtration* to $\upgamma$ at *p*. If $\upgamma$ is Legendrian, then $\mathrm {D}$ is a Lagrangian subspace and $\mathrm {H}$ is the symplectic orthogonal to $\ell$. Since $\mathrm {GL}(2,\mathbb {R})$ acts transitively on $\upgamma$, we may choose $\ell$ to be spanned by the first basis vector $e_1\odot e_1\odot e_1$. Its stabilizer

2.7 $$\begin{aligned} B:=\{g\in \mathrm {GL}(2,\mathbb {R}): \phi (g)(\ell )\subset \ell \} \end{aligned}$$

is given by those matrices in (2.4) for which the parameter $\rho =0$. The block form of *B* reflects the fact that *B* preserves the filtration (2.6).

#### Remark 1

The filtration (2.6) is also visible in the $\mathrm {G}_2$ root diagram. Let the line $\ell \subset \mathfrak {g}_{-1}$ be spanned by $E_4$. The subalgebra of $\mathfrak {g}_0\cong \mathfrak {gl}(2,\mathbb {R})$ that preserves this line via the adjoint representation on $\mathfrak {g}_{-1}$ is $\mathfrak {b}=\mathrm {span}(E_5, E_6, E_8)$. It is visible that this subalgebra preserves the filtration

$$\begin{aligned} \mathrm {span}(E_4)\subset \mathrm {span}(E_4,E_3)\subset \mathrm {span}(E_4, E_3,E_2)\subset \mathrm {span}(E_4,E_3,E_2,E_1)=\mathfrak {g}_{-1}. \end{aligned}$$

Applying the algebraic observations point by point to $\upgamma ={\varvec{\upgamma }}_x\subset \mathcal {C}_x$ and $p=\sigma (x),$ $x\in \mathcal {U}$, gives rise to the following proposition.

#### Proposition 1

A marked contact Engel structure $(\mathcal {U},\mathcal {C},{\varvec{\upgamma }},\sigma )$ is equipped with a flag of distributions

2.8 $$\begin{aligned} \ell ^{\sigma }\subset \mathcal {D}^{\sigma }\subset \mathcal {H}^{\sigma }\subset \mathcal {C}\subset T\mathcal {U}, \end{aligned}$$

where the rank 2 distribution $\mathcal {D}^{\sigma }\subset \mathcal {C}$ is Legendrian (i.e., totally null with respect to the conformal symplectic structure on $\mathcal {C}$) and the rank 3 distribution $\mathcal {H}^{\sigma }$ is the symplectic orthogonal to $\ell ^{\sigma }$.

#### Definition 2

A marked contact Engel structure is called *integrable* if the rank two distribution $\mathcal {D}^{\sigma }$ is integrable. In this case $\sigma$ is called an *integrable section*.

### A Convenient Coordinate Representation

A convenient way to represent a marked contact Engel structure is in terms of a smooth function $t=t(x^0, x^1, x^2, x^3, x^4)$, where $\{x^i\}$ are coordinates on $\mathcal {U}$ as in Sect. 1. Let $(X_0, X_1, X_2, X_3, X_4)$ denote the frame (1.3) dual to $(\alpha ^0,\alpha ^1,\alpha ^2,\alpha ^3,\alpha ^4)$ as in (1.2). We may assume that the section $\sigma :\mathcal {U}\rightarrow {\varvec{\upgamma }}$ defining the *marked* contact Engel structure is of the form

2.9 $$\begin{aligned} \sigma =[-t^3 X_1+t^2 X_2-t X_3+ X_4], \end{aligned}$$

for a smooth function $t=t(x^0, x^1, x^2, x^3, x^4)$.^3^ Then the filtration from Proposition 1 is of the form

2.10 $$\begin{aligned} \ell ^{\sigma }=\mathrm {Span}(\xi _4)\subset \mathcal {D}^{\sigma }=\mathrm {Span} (\xi _4,\xi _3)\subset \mathcal {H}^{\sigma }&=\mathrm {Span}(\xi _4,\xi _3,\xi _2)\nonumber \\&\subset \mathcal {C}=\mathrm {Span}(\xi _4,\xi _3,\xi _2,\xi _1), \end{aligned}$$

where

2.11 $$\begin{aligned} \begin{array}{l} \xi _0:= X_0=\, \partial _{x^0}\\ \xi _1:=X_1=\,\partial _{x^1} \\ \xi _2:=-3 t X_1+ X_2=\, -3 t \partial _{x^1}+\partial _{x^2}\\ \xi _3:= 3 t^2 X_1-2 t X_2+ X_3=\, 3x^2\partial _{x^0}+3t^2\partial _{x^1} -2t\partial _{x^2}+\partial _{x^3} \\ \xi _4:=-t^3 X_1+t^2 X_2-t X_3+ X_4=\, -(x^1+3tx^2)\partial _{x^0}-t^3 \partial _{x^1}\\ \qquad \qquad +\,t^2\partial _{x^2}-t\partial _{x^3}+\partial _{x^4} \,. \end{array} \end{aligned}$$

The coframe dual to the frame $(\xi _0, \xi _1, \xi _2, \xi _3, \xi _4)$ is of the form

2.12 $$\begin{aligned} \begin{pmatrix} \omega ^0\\ \omega ^1\\ \omega ^2\\ \omega ^3\\ \omega ^4\\ \end{pmatrix}= \begin{pmatrix}\begin{aligned} &{}\mathrm{d}x^0+x^1\mathrm{d}x^4- 3 x^2\mathrm{d}x^3\\ &{}\mathrm{d}x^1+3 t\mathrm{d}x^2+ 3 t^2 \mathrm{d}x^3+t^3\mathrm{d}x^4\\ &{}\mathrm{d}x^2 +2 t \mathrm{d}x^3 +t^2 \mathrm{d}x^4\\ &{}\mathrm{d}x^3+ t \mathrm{d}x^4\\ &{}\mathrm{d}x^4\\ \end{aligned} \end{pmatrix}. \end{aligned}$$

The osculating filtration (2.10) is given in terms of this coframe as

2.13 $$\begin{aligned} \ell ^{\sigma }&=\mathrm {ker}(\omega ^0,\omega ^1,\omega ^2,\omega ^3) \subset \mathcal {D}^{\sigma }=\mathrm {ker}(\omega ^0,\omega ^1,\omega ^2) \subset \mathcal {H}^{\sigma }=\mathrm {ker}(\omega ^0,\omega ^1)\nonumber \\&\subset \mathcal {C}=\mathrm {ker}(\omega ^0). \end{aligned}$$

#### Proposition 2

The marked contact Engel structure represented by *t* is integrable if and only if

$$\begin{aligned} \mathcal {J}=(x^1+3tx^2)t_{x^0}+t^3 t_{x^1} - t^2 t_{x^2} + t t_{x^3} -t_{x^4}=0, \quad \hbox {where}\quad t_{x^i}=\partial _{x^i}t. \end{aligned}$$

#### Proof

A straightforward calculation gives $\mathrm{d}\omega ^i\wedge \omega ^0\wedge \omega ^1\wedge \omega ^2=0$, for $i=0,1$, and $\mathrm{d}\omega ^2\wedge \omega ^0\wedge \omega ^1\wedge \omega ^2= 2 \ \mathcal {J}\; \omega ^0\wedge \omega ^1\wedge \omega ^2\wedge \omega ^3\wedge \omega ^4$. $\square$

### The $\mathrm {G}_2$-Double Fibration and a Kerr Theorem

The integrability condition introduced in Definition 2 is analogous to a theorem from Relativity attributed to Kerr [14, 17], as the following theorem shows.^4^

#### Theorem 1

(Kerr theorem for contact Engel structures) The general smooth solution to the equation

2.14 $$\begin{aligned} \mathcal {J}=(x^1+3tx^2)t_{x^0}+t^3 t_{x^1} - t^2 t_{x^2} + t t_{x^3} -t_{x^4}=0 \end{aligned}$$

is obtainable locally by choosing an arbitrary smooth function *F* of five variables and solving, for *t* in terms of $x^0, x^1, x^2, x^3, x^4$, the equation

$$\begin{aligned} F(x^0+x^1x^4+3tx^2x^4-t^3(x^4)^2, x^1+t^3x^4, x^2-t^2x^4, x^3+t x^4, t)=0\,. \end{aligned}$$

#### Proof

We introduce the following variables

2.15 $$\begin{aligned} y^0=x^0+x^1x^4+3t x^2x^4-t^3(x^4)^2, \quad y^1&=x^1+t^3x^4,\quad y^2=x^2-t^2x^4,\nonumber \\&y^3=x^3+tx^4. \end{aligned}$$

Then $\mathrm{d}\omega ^0\wedge \omega ^0\wedge \omega ^1\wedge \omega ^2=0,$ $\mathrm{d}\omega ^1\wedge \omega ^0\wedge \omega ^1\wedge \omega ^2=0$ and in the new variables we have $\mathrm{d}\omega ^2\wedge \omega ^0\wedge \omega ^1\wedge \omega ^2= -2 \; \mathrm{d}t\wedge \mathrm{d}y^0\wedge \mathrm{d}y^1\wedge \mathrm{d}y^2\wedge \mathrm{d}y^3.$ The latter expression vanishes if and only if there exists a smooth function *F* of five variables such that $F(t, y^0,y^1,y^2,y^3)=0$. On the other hand, vanishing of $\mathrm{d}\omega ^2\wedge \omega ^0\wedge \omega ^1\wedge \omega ^2$ is equivalent to integrability of $\mathcal {D}^{\sigma },$ which we have seen is equivalent to $\mathcal {J}=0$ in Proposition 2. $\square$

Similarly to the classical Kerr Theorem, also Theorem 1 can be understood in terms of a *twistorial correspondence*. The two 5-dimensional homogeneous spaces of $\mathrm {G}_2$ are related by a *double fibration* of the following form. Here $\mathrm {P}_{1,2}=\mathrm {P}_1\cap \mathrm {P}_2$ is the 8-dimensional Borel subgroup of $\mathrm {G}_2$.


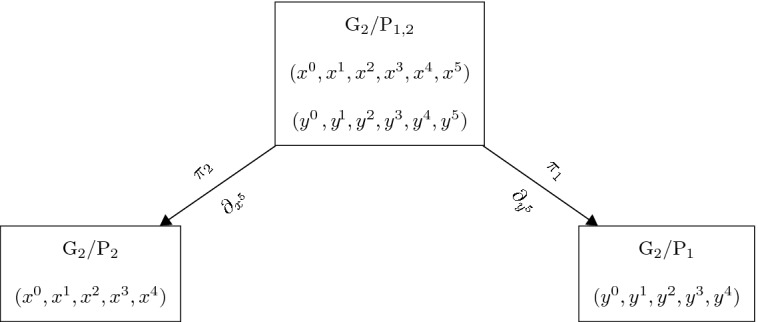


The total space ${\varvec{\upgamma }}\subset {\mathbb {P}}(\mathcal {C})$ of the twisted cubic bundle can be identified with the 6-dimensional homogeneous space ${\mathrm {G}}_2/P_{1,2}$ (see [9] for details). Thus, *a marked contact Engel structure is given by an open subset* $\mathcal {U}\subset \mathrm {G}_2/P_2$ *together with a smooth section* $\sigma$ *of* $\pi _2\vert _{\mathcal {U}}$,

$$\begin{aligned} \mathrm {G}_2/\mathrm {P}_2\supset \mathcal {U}\xrightarrow {\sigma } \sigma (\mathcal {U})\subset \mathrm {G}_2/\mathrm {P}_{1,2}. \end{aligned}$$

We now consider two sets of local coordinates $\{x^i\}_{i=1,\dots ,5}$ and $\{y^i\}_{i=1,\dots ,5}$ on $\mathrm {G}_2/\mathrm {P}_{1,2}$ adapted to the two legs of the double fibration. In terms of $\{x^i\}_{i=1,\dots ,5}$, we have

$$\begin{aligned} \pi _2:(x^0,x^1,x^2,x^3,x^4, x^5)\mapsto (x^0,x^1,x^2,x^3,x^4), \end{aligned}$$

where $\{x^i\}_{i=1,\dots ,4}$ are coordinates on $\mathcal {U}\subset \mathrm {G}_2/P_2$ as introduced in Sect. 1. The vertical bundle for $\pi _2$ is spanned by $\xi _7=-\partial _{x^5}$ and the vertical bundle for $\pi _1$ is spanned by

$$\begin{aligned} \xi _4=-(x^1+3x^5x^2)\partial _{x^0}-(x^5)^3\partial _{x^1}+(x^5)^2\partial _{x^2}-(x^5) \partial _{x^3}+\partial _{x^4}. \end{aligned}$$

In terms of $\{y^i\}_{i=1,\dots ,5}$, the field $\xi _4$ spanning the vertical bundle for $\pi _1$ is rectified, i.e., $\xi _4=\partial _{y^5},$ and $\xi _7=-3 y^5 y^2 \partial _{y^0}-3 (y^4)^2 y^5 \partial _{y^1}+2 y^4 y^5 \partial _{y^2}-y^5 \partial _{y^3}-\partial _{y^4}\, .$

The coordinate systems are related by

2.16 $$\begin{aligned}&y^0=x^0+x^1x^4+3x^5 x^2x^4-(x^5)^3(x^4)^2, \quad y^1=x^1+(x^5)^3x^4,\nonumber \\&y^2=x^2-(x^5)^2x^4,\quad y^3=x^3+x^5x^4,\quad y^4=x^5, \quad y^5=x^4. \end{aligned}$$

The geometrical interpretation of Theorem 1 is now almost immediate.

#### Corollary 1

There is a local bijective correspondence between integrable sections of $\pi _2$ and hypersurfaces $\Sigma \subset \mathrm {G}_2/\mathrm {P}_1$ that are generic in the sense that their preimages ${\pi _1}^{-1}(\Sigma )$ intersect the fibres ${\pi _2}^{-1}(x)$ transversally.

#### Proof

A section $\sigma :\mathcal {U}\rightarrow \mathrm {G}_2/\mathrm {P}_{1,2}$, represented by a function $t\in C^{\infty }(\mathcal {U})$ on $\mathcal {U}\subset \mathrm {G}_2/\mathrm {P}_2,$ defines a hypersurface in $\mathrm {G}_2/\mathrm {P}_{1,2}$, given

by its graph $x^5=t(x^0,x^1,x^2,x^3,x^4).$ By Proposition 2, the condition that $\sigma$ be integrable reads

$$\begin{aligned} 0=-(x^1+3tx^2)t_{x^0}-t^3 t_{x^1}+t^2 t_{x^2}-t t_{x^3}+ t_{x^4}=\xi _4(t)\vert _{\sigma (\mathcal {U})}. \end{aligned}$$

Since $\xi _4$ spans the vertical bundle of $\pi _1$, this means that $\sigma (\mathcal {U})$ is tangential to the fibres of $\pi _1$, which implies that $\sigma$ defines a hypersurface in $\mathrm {G}_2/\mathrm {P}_1$.

Conversely, let $\Sigma$ be a hypersurface in $\mathrm {G}_2/\mathrm {P}_1$ such that $\pi _1^{-1}(\Sigma )$ is transversal to the fibres of $\pi _2$.

Because of this genericity assumption on $\Sigma$, we may apply the implicit function theorem and write $\pi _1^{-1}(\Sigma )$, locally, as the graph of a section $x^5=t(x^0,x^1,x^2,x^3,x^4)$. By construction , i.e., the section is integrable. $\square$

### Maximal and Submaximal Models

The correspondence from Corollary 1 gives rise to geometric descriptions of the most symmetrical marked contact Engel structures.

It is well known that $\mathrm {G}_2$ can be realized as the subgroup in $\mathrm {GL}(7,\mathbb {R})$ that fixes a generic three-form $\Phi \in \Lambda ^3(\mathbb {R}^{7})^*$. (There are two open $\mathrm {GL}(7,\mathbb {R})$-orbits in $\Lambda ^3(\mathbb {R}^{7})^*$ with stabilizer subgroups the split real form and compact real form of the complex group ${\mathrm {G}_2}^{\mathbb {C}}$ respectively.) The three-form $\Phi$ determines a $\mathrm {G_2}$-invariant bilinear form $h\in \bigodot ^2(\mathbb {R}^{7})^*$ of signature (3, 4). The homogeneous spaces occurring in the $\mathrm {G}_2$-double fibration admit the following descriptions (see e.g. [2, 8] for details):


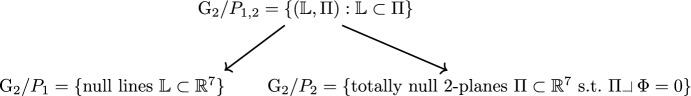


A fibre ${\pi _2}^{-1}(\Pi )$ can be identified with the set of all 1-dimensional subspaces contained in $\Pi$ and is thus isomorphic to $\mathbb {R}\mathbb {P}^1$. A fibre ${\pi _1}^{-1}(\mathbb {L})$ can be identified with the set of all totally null 2-dimensional subspaces $\Pi$ that insert trivially into $\Phi$ and contain $\mathbb {L}$; this is the set of 2-dimensional subspaces of the 3-dimensional null subspace , and hence also isomorphic to $\mathbb {R}\mathbb {P}^1$.

Viewing $\mathrm {G}_2/\mathrm {P}_1$ as a projectivized null cone, the simplest kinds of hypersurfaces in $\mathrm {G}_2/\mathrm {P}_1$ are obtained by intersecting the null cone with a 6-dimensional vector subspace $\mathbb {W}\subset \mathbb {R}^7$ and projectivizing. Such hyperplanes $\mathbb {W}=\mathbb {L}^{\perp }$ split into three classes according to whether its annihilator $\mathbb {L}$ is a lightlike, timelike or spacelike line with respect to *h*. It is further known that the group $\mathrm {G}_2$ acts transitively on the set of, respectively, lightlike, timelike, spacelike lines $\mathbb {L}\subset \mathbb {R}^{7}$ and that

- $$\mathrm {Stab}_{\mathrm {G}_2}(\mathbb {L})=\mathrm {P}_1$$ iff $$\left\langle \mathbb {L},\mathbb {L}\right\rangle =0$$,
- $$\mathrm {Stab}_{\mathrm {G}_2}(\mathbb {L})=\mathrm {SU}(1,2)$$ iff $$\left\langle \mathbb {L},\mathbb {L}\right\rangle >0$$,
- $$\mathrm {Stab}_{\mathrm {G}_2}(\mathbb {L})=\mathrm {SL}(3,\mathbb {R})$$ iff $$\left\langle \mathbb {L},\mathbb {L}\right\rangle <0$$.

Each of these groups has a unique open orbit $\Sigma _{\mathbb {L}}\subset \mathrm {G}_2/P_1$, see [16].

According to Corollary 1, there are corresponding marked contact Engel structures: These are defined on the subsets ${\mathcal M}_{\mathbb {L}}:=\{ \Pi \in \mathrm {G}_2/\mathrm {P}_2\mid \dim (\Pi \cap \mathbb {L}^\perp )=1\}\subset \mathrm {G}_2/\mathrm {P}_2.$ The section $\sigma$ given by

2.17 $$\begin{aligned} \sigma (\Pi ):=( \Pi ,\Pi \cap \mathbb {L}^\perp )\in \mathrm {G}_2/\mathrm {P}_{1,2}\, . \end{aligned}$$

equips such a subset with a $\mathrm {Stab}_{\mathrm {G}_2}(\mathbb {L})$-invariant marked contact Engel structure. It follows from the analysis of the next section that the symmetry algebras of these structures can’t be bigger, so they are exactly $\mathfrak {p}_1$, $\mathfrak {sl}(3,\mathbb {R})$, and $\mathfrak {su}(1,2)$, respectively (note that these are maximal subalgebras of the Lie algebra of $\mathrm {G}_2$).

Utilizing Theorem 1, a function *t* locally defining all these three structures can be chosen to be $t=\frac{x^1-\epsilon x^3}{-x^2+\epsilon x^4}$. Here $\epsilon =0,\pm 1$, and *t* with $\epsilon =0$ corresponds to the marked contact Engel structure with $\mathfrak {p}_1$ symmetry, and functions *t* with $\epsilon =1$ or $\epsilon =-1$ correspond to the two nonequivalent models with 8-dimensional symmetry algebras.

## Local Invariants and Homogeneous Models of Marked Contact Engel Structures via Cartan’s Equivalence Method

In order to obtain a complete picture of the local invariants of marked contact Engel structures, we now apply Cartan’s method (see e.g. [13] for an introduction) to the local equivalence problem of these structures. In particular, we obtain a classification of all homogeneous marked contact Engel structures with symmetry algebras of dimension $\ge 6$ up to local equivalence. Additional computational details are provided in the arXiv version [9] of the article.

### Adapted Coframes

Let $(\mathcal {U},\mathcal {C},{\varvec{\upgamma }},\sigma )$ be a marked contact Engel structure. In order to apply Cartan’s equivalence method, we need to formulate the notion of (local) equivalence of two such structures in terms of adapted coframes.

#### Definition 3

A (local) coframe $\mathbf {\omega }=(\omega ^0,\omega ^1,\omega ^2,\omega ^3,\omega ^4)$ is called 0-*adapted* to the marked contact Engel structure $(\mathcal {U},\mathcal {C},{\varvec{\upgamma }},\sigma )$ if and only if

1. the 1-form $\omega ^0$ is a contact form such that
  $$\begin{aligned} \mathcal {C}=\mathrm {ker}(\omega ^0), \end{aligned}$$
2. ${\varvec{\upgamma }}\subset \mathbb {P}(\mathcal {C})$ is the projectivization of the set of all tangent vectors contained in $\mathcal {C}$ that are simultaneously null for the three symmetric tensor fields
  $$\begin{aligned} g_1=\omega ^1\omega ^3- (\omega ^2)^2,\quad g_2= \omega ^2\omega ^4- (\omega ^3)^2,\quad g_3= \omega ^2\omega ^3-\omega ^1\omega ^4, \end{aligned}$$
3. the line field $\ell ^{\sigma }$ is given by
  $$\begin{aligned} \ell ^{\sigma } =\mathrm {ker} (\omega ^0,\omega ^1, \omega ^2, \omega ^3). \end{aligned}$$

#### Definition 4

Consider two marked contact Engel structures $(\mathcal {U},\mathcal {C},{\varvec{\upgamma }},\sigma )$ and $(\bar{\mathcal {U}},\bar{\mathcal {C}}, {\bar{\varvec{\upgamma }}},\bar{\sigma })$. Let ${\omega }$ and ${\bar{\omega }}$ be 0-adapted coframes of the respective structures. Then the two marked contact Engel structures are (locally) equivalent if there exists a (local) diffeomorphism $f:\mathcal {U}\rightarrow \bar{\mathcal {U}}$ and a function $A=(A^{i}{}_{j}):\mathcal {U}\rightarrow \mathbf {A}$ taking values in the group

3.1 $$\begin{aligned} \mathbf {A}=\left\{ \begin{pmatrix} s_0&{} 0&{}0&{}0&{}0\\ s_1&{}{s_5}^3&{} 0 &{} 0&{}0\\ s_2&{}{s_5}^2s_7&{} {s_5}^2 s_8&{} 0 &{}0\\ s_3&{} s_5 {s_7}^2&{} 2 s_7 s_5 s_8 &{} s_5{s_8}^2&{} 0\\ s_4&{} {s_7}^3&{} 3 {s_7}^2s_8&{}3s_7{s_8}^2&{}{s_8}^3 \end{pmatrix} :\, s_0 {s_5}^6{s_8}^6\ne 0\right\} , \end{aligned}$$

such that $f^*\bar{\omega }^i=A^i_j\omega ^j$. The group $\mathbf {A}$ is called the structure group for marked contact Engel structures.

The bottom right $4\times 4$ block matrices in *A* form a group isomorphic to the Borel subgroup $B\subset \mathrm {GL}(2,\mathbb {R})$, defined in (2.7), in the irreducible representation (2.4). In particular, $\mathbf {A}\cong B < imes \mathbb {R}^5$.

We proceed with a number of technical, but important lemmas. Consider the most general marked contact Engel structure locally represented by a smooth function $t=t(x^0, x^1, x^2, x^3, x^4)\in C^{\infty }(\mathcal {U})$ and its 0-adapted coframe (2.12). Differentiating the coframe and then expanding $\mathrm{d}t$ in terms of the coframe we obtain that the coframe (2.12) moreover satisfies structure equations of the below form (3.2), which shows that:

#### Lemma 1

Any marked contact Engel structure admits a 0-adapted coframe $(\omega ^0,\omega ^1,\omega ^2,\omega ^3,\omega ^4)$ satisfying

3.2 $$\begin{aligned} \begin{array}{llll} \mathrm{d}\omega ^0=\omega ^1\wedge \omega ^4-3\omega ^2\wedge \omega ^3\\ \mathrm{d}\omega ^1=\tfrac{3}{4}(b^2-4ac+M-P)\omega ^0\wedge \omega ^2+3c\omega ^1\wedge \omega ^2-3 a \omega ^2\wedge \omega ^3 +3 J \omega ^2\wedge \omega ^4\\ \mathrm{d}\omega ^2= \tfrac{1}{2}(b^2-4ac+M-P) \omega ^0\wedge \omega ^3+2 c\omega ^1\wedge \omega ^3-2 b\omega ^2\wedge \omega ^3+2 J \omega ^3\wedge \omega ^4\\ \mathrm{d}\omega ^3=\tfrac{1}{4}(b^2-4ac+M-P)\omega ^0\wedge \omega ^4+c\omega ^1\wedge \omega ^4-b\omega ^2\wedge \omega ^4+a\omega ^3\wedge \omega ^4\\ \mathrm{d}\omega ^4=0\\ \end{array} \end{aligned}$$

for functions *a*, *b*, *c*, *J*, *M*, *P* on $\mathcal {U}$.

#### Definition 5

A coframe as in Lemma 1 is called 1-adapted.

Applying the exterior derivative on both sides of (3.2) we get information about the exterior derivatives of the functions *a*, *b*, *c* and *J*. A subscript $\omega ^i$ denotes the *i*th frame derivative, i.e., $\mathrm {d}F=F_{\omega ^i}\omega ^i$.

#### Lemma 2

The functions *a*, *b*, *c* and *J* from Lemma 1 satisfy

3.3 $$\begin{aligned} \begin{array}{l} \mathrm{d}J=J_{\omega ^0}\omega ^0+J_{\omega ^1}\omega ^1+J_{\omega ^2}\omega ^2+J_{\omega ^3}\omega ^3+J_{\omega ^4}\omega ^4\\ \mathrm{d}a=a_{\omega ^0}\omega ^0+a_{\omega ^1}\omega ^1+\tfrac{1}{4}(-3b^2+M+3P)\omega ^2+L\omega ^3+(a^2-2bJ-J_{\omega ^3})\omega ^4\\ \mathrm{d}b=\tfrac{1}{4}(-4 a_{\omega ^1} b + 6 b^2 c - 8 a c^2 + 4 c M - M_{\omega ^2} + P_{\omega ^2} + 2 b Q - 4 a R)\omega ^0\\ \quad \qquad +(2c^2+R)\omega ^1 +\,(2 a_{\omega ^1}-3bc-Q)\omega ^2+\tfrac{1}{2}(-b^2+M-3P)\omega ^3\\ \quad \qquad +(ab-3cJ+J_{\omega ^2})\omega ^4\\ \mathrm{d}c =c_{\omega ^0}\omega ^0+S\omega ^1+(c^2-R)\omega ^2+(a_{\omega ^1}-2bc)\omega ^3\\ \quad \qquad +\,\tfrac{1}{4}(b^2-4J_{\omega ^1}+M-P)\omega ^4 , \end{array} \end{aligned}$$

for functions *L*, *Q*, *R*, *S* on $\mathcal {U}$.

#### Lemma 3

The functions *a*, *b*, *c*, *J*, *L*, *M*, *P*, *Q*, *R*, *S* are uniquely determined by (3.2) and (3.3). Explicitly, for a marked contact Engel structure determined by a function $t\in C^{\infty }(\mathcal {U})$,

$$\begin{aligned}&a= t_{\omega ^3}, \quad b=-t_{\omega ^2},\quad c=t_{\omega ^1},\quad J=-t_{\omega ^4},\quad L=t_{\omega ^3\omega ^3},\quad M=6t_{\omega ^0}\\&-2(t_{\omega ^2})^2+6 t_{\omega ^3} t_{\omega ^1}+ t_{\omega ^2\omega ^3},\\&P=2t_{\omega ^0}-(t_{\omega ^2})^2+2 t_{\omega ^3} t_{\omega ^1}+t_{\omega ^2\omega ^3},\quad Q=2 t_{\omega ^3\omega ^1} \\&+t_{\omega ^2\omega ^2}+3t_{\omega ^2} t_{\omega ^1},\quad R=-t_{\omega ^2\omega ^1}-2(t_{\omega ^1})^2,\quad S=t_{\omega ^1\omega ^1}. \end{aligned}$$

### The Associated Invariant Coframe

Our next goal is to construct an invariant coframe (i.e., an *e*-structure) on a 9-dimensional bundle associated with any marked contact Engel structure.

We start by choosing a 1-adapted coframe $\mathbf {\omega }$

and we lift it to the 5 well-defined (tautological) 1-forms

$$\begin{aligned} \theta ^{i}=\mathbf {A}^{i}{}_{j}\omega ^{j},\quad i=0,1,2,3,4, \end{aligned}$$

on $\mathcal {U}\times \mathbf {A}$, where $\mathbf {A}$ is the structure group (3.1). Writing equations (3.2) symbolically as

3.4 $$\begin{aligned} \mathrm{d}\omega ^{i}=-\tfrac{1}{2}F^{i}{}_{jk}\omega ^{j}\wedge \omega ^{k}, \end{aligned}$$

we express the differentials $\mathrm{d}\theta ^0,...,\mathrm{d}\theta ^4$ as

$$\begin{aligned} \mathrm{d}\theta ^{i}&=\mathrm{d}(\mathbf {A}^{i}{}_{j}\omega ^{j})=\mathrm{d}\mathbf {A}^{i}{}_{j}\wedge \omega ^{j}+ \mathbf {A}^{i}{}_{j}\mathrm{d}\omega ^{j}\\&=\mathrm{d}\mathbf {A}^{i}{}_{k}(\mathbf {A}^{-1})^{k}{}_{l}\wedge \theta ^{l}-\tfrac{1}{2}\mathbf {A}^{i}{}_{j} F^{j}{}_{kl}(\mathbf {A}^{-1})^{k}{}_{n}(\mathbf {A}^{-1})^{l}{}_{m}\theta ^{n}\wedge \theta ^{m}. \end{aligned}$$

For computational reasons we set $\delta =-s_5 s_8.$

Since $\ \mathrm{d}\theta ^0 \wedge \theta ^0=- \tfrac{s_0}{\delta ^3}\theta ^1\wedge \theta ^4\wedge \theta ^0 +\tfrac{3 s_0}{\delta ^3}\theta ^2\wedge \theta ^3\wedge \theta ^0,$ we can normalize the coefficient of the $\theta ^1\wedge \theta ^4$–term in the expansion of $\mathrm{d}\theta ^0$ to 1 by setting

3.5 $$\begin{aligned} s_0=-{\delta ^3}. \end{aligned}$$

Then there exists a 1-form $\theta ^5$, which is uniquely defined up to addition of multiples of $\theta ^0$, satisfying

$$\begin{aligned} \mathrm{d}\theta ^0= -6 \theta ^0\wedge \theta ^5 + \theta ^1\wedge \theta ^4 - 3 \theta ^2\wedge \theta ^3. \end{aligned}$$

Computing $$\ \mathrm{d}\theta ^1 \wedge \theta ^0\wedge \theta ^1\wedge \theta ^4=\tfrac{3(s_1\delta +a {s_5}^3\delta -3 J{s_5}^4{s_7})}{{\delta }^4}\theta ^0\wedge \theta ^1\wedge \theta ^2\wedge \theta ^3\wedge \theta ^4\ $$ shows that we can further normalize the $\theta ^2\wedge \theta ^3$–coefficient in the expansion of $\mathrm{d}\theta ^1$ to 0 by setting

3.6 $$\begin{aligned} s_1=\tfrac{-a \delta {s_5}^3+3 J {s_5}^4{s_7}}{\delta }. \end{aligned}$$

Then there exists a 1-form $\theta ^8$, uniquely defined up to addition of multiples of $\theta ^0$ and $\theta ^1$, satisfying

$$\begin{aligned} \mathrm{d}\theta ^1\wedge \theta ^0=-3 \theta ^0\wedge \theta ^1\wedge \theta ^5-3 \theta ^0\wedge \theta ^1\wedge \theta ^8+\tfrac{3 J {s_5}^5}{\delta ^4}\theta ^0\wedge \theta ^2\wedge \theta ^4. \end{aligned}$$

Now $\mathrm{d}\theta ^2\wedge \theta ^0\wedge \theta ^1= \tfrac{2(2 \delta s_2-b\delta ^2 s_5+2 a \delta {s_5}^2s_7-3J {s_5}^3{s_7}^2)}{\delta ^4}\theta ^0\wedge \theta ^1\wedge \theta ^2\wedge \theta ^3-3 \theta ^0\wedge \theta ^1\wedge \theta ^2\wedge \theta ^5 -\theta ^0\wedge \theta ^1\wedge \theta ^2\wedge \theta ^8 +\tfrac{2 J{s_5}^5}{\delta ^4}\theta ^0\wedge \theta ^1\wedge \theta ^3\wedge \theta ^4$ shows that we can normalize the $\theta ^2\wedge \theta ^3$–term in the expansion of $\mathrm{d}\theta ^2$ to 0 by setting

3.7 $$\begin{aligned} s_2=\tfrac{s_5}{2\delta }(b\delta ^2-2a\delta s_5 s_7+3J{s_5}^2{s_7}^2), \end{aligned}$$

and $$\ \mathrm{d}\theta ^3\wedge \theta ^0\wedge \theta ^2\wedge \theta ^3 = -\tfrac{c \delta ^3+\delta s_3 s_5 - b \delta ^2 s_5 s_7 + a\delta {s_5}^2 {s_7}^2-J{s_5}^3{s_7}^3}{\delta ^4 s_5} \theta ^0\wedge \theta ^1\wedge \theta ^2\wedge \theta ^3\wedge \theta ^4\ $$ shows that we can normalize the $\theta ^1\wedge \theta ^4$–term in the expansion of $\mathrm{d}\theta ^3$ to 0 by setting

3.8 $$\begin{aligned} s_3=-\tfrac{1}{\delta s_5}(c\delta ^3 - b \delta ^2 s_5 {s_7}+a\delta {s_5}^2{s_7}^2-J {s_5}^3{s_7}^3). \end{aligned}$$

Having performed these normalizations, on $\mathcal {G}^9\subset (\mathcal {U}\times \mathbf {A})$ defined by (3.5), (3.6), (3.7), (3.8), we now have

3.9 $$\begin{aligned}&\theta ^0=-\delta ^3\omega ^0\nonumber \\&\theta ^1=\tfrac{{s_5}^3(3J {s_5}{s_7}-a\delta )}{\delta }\omega ^0+{s_5}^3\omega ^1\nonumber \\&\theta ^2=\tfrac{s_5(b\delta ^2 - 2 a\delta s_5 s_7+3 J {s_5}^2{s_7}^2)}{2\delta }\omega ^0+{s_5}^2 s_7\omega ^1-\delta s_5\omega ^2\nonumber \\&\theta ^3=\tfrac{-c\delta ^3+b\delta ^2 s_5 s_7- a\delta {s_5}^2{s_7}^2+J{s_5}^3{s_7}^3}{s_5}\omega ^0+s_5{s_7}^2\omega ^1-2 \delta s_7 \omega ^2 +\tfrac{\delta ^2}{s_5}\omega ^3\nonumber \\&\theta ^4=s_4\omega ^0+{s_7}^3\omega ^1-\tfrac{3\delta {s_7}^2}{s_5}\omega ^2+\tfrac{3\delta ^2s_7}{{s_5}^2}\omega ^3-\tfrac{\delta ^3}{{s_5}^3}\omega ^4. \end{aligned}$$

We have further introduced two additional forms $\theta ^5$ and $\theta ^8$, but on the 9-dimensional bundle $\mathcal {G}^9$ given by (3.5), (3.6), (3.7), (3.8) they are defined up to a certain freedom. It turns out that imposing further normalizations determines forms $\theta ^5$, $\theta ^8$ uniquely and in addition picks up unique 1-forms $\theta ^6$ and $\theta ^{12}$ that together with the five 1-forms (3.9) constitute a coframe on $\mathcal {G}^9$. The normalizations needed are included in the following proposition^5^:

#### Proposition 3

The five forms (3.9) on the 9-dimensional subbundle $\mathcal {G}^9\subset \mathcal {U}\times \mathbf {A}$ given by (3.5), (3.6), (3.7), (3.8) can be supplemented to an invariant coframe $(\theta ^0, \theta ^1, \theta ^2, \theta ^3, \theta ^4, \theta ^5, \theta ^6, \theta ^8, \theta ^{12})$, which is uniquely determined by the fact that it satisfies

3.10 $$\begin{aligned} \mathrm{d}\theta ^0&\,=\,-6\theta ^0\wedge \theta ^5+\theta ^1\wedge \theta ^4-3\theta ^2\wedge \theta ^3\nonumber \\ \mathrm{d}\theta ^1&\,=\, - 3 \theta ^1\wedge \theta ^5 - 3 \theta ^1\wedge \theta ^8+ T^1{}_{02} \theta ^0\wedge \theta ^2 + T^1{}_{03} \theta ^0\wedge \theta ^3 + T^1{}_{04} \theta ^0\wedge \theta ^4\nonumber \\&\qquad \qquad + T^1{}_{06} \theta ^0\wedge \theta ^6 + T^1{}_{24}\theta ^2\wedge \theta ^4\nonumber \\ \mathrm{d}\theta ^2&\,=\, \theta ^1\wedge \theta ^6- 3\theta ^2\wedge \theta ^5 - \theta ^2\wedge \theta ^8 + T^2{}_{03} \theta ^0\wedge \theta ^3 - T^2{}_{04} \theta ^0\wedge \theta ^4 +T^2{}_{34}\theta ^3\wedge \theta ^4\nonumber \\ \mathrm{d}\theta ^3&\,=\,2 \theta ^2\wedge \theta ^6 - 3\theta ^3\wedge \theta ^5 + \theta ^3\wedge \theta ^8+T^3{}_{01}\theta ^0\wedge \theta ^1 \nonumber \\&\qquad \qquad + T^3{}_{02} \theta ^0\wedge \theta ^2 - T^3{}_{03} \theta ^0\wedge \theta ^3 + T^3{}_{04} \theta ^0\wedge \theta ^4\nonumber \\ \mathrm{d}\theta ^4&\,=\,6 \theta ^0\wedge \theta ^{12}+3\theta ^3\wedge \theta ^6-3\theta ^4\wedge \theta ^5+3\theta ^4\wedge \theta ^8, \end{aligned}$$

for some functions $T^i{}_{jk}$, and the additional normalization that $\mathrm{d}\theta ^5$, when written with respect to the basis of forms $\theta ^i\wedge \theta ^j$, has zero coefficient at the $\theta ^0\wedge \theta ^1$ term. We have

3.11 $$\begin{aligned} T^1{}_{24}=-T^1{}_{06}=\tfrac{3}{2}T^2{}_{34}=\tfrac{3 J {s_5}^5}{\delta ^4}. \end{aligned}$$

In particular, *J* is a relative invariant for marked contact Engel structures.

### Integrable Structures (the $J=0$ Case)

The geometric interpretation of the $J=0$ condition is immediately visible from the structure equations (3.10). It means that the contact Engel structure is integrable, see Definition 2 and Proposition 2. (In the representation of a marked contact Engel structure given by a function $t\in C^{\infty }(\mathcal {U})$, the function *J* coincides with $\mathcal {J}$ introduced in Proposition 2.)

For integrable structures, the structure equations of the invariant coframe from Proposition 3 simplify, and the first five read as follows:

3.12 $$\begin{aligned} \mathrm{d}\theta ^0&\,=\,-6\theta ^0\wedge \theta ^5+\theta ^1\wedge \theta ^4-3\theta ^2\wedge \theta ^3\nonumber \\ \mathrm{d}\theta ^1&\,=\,-3 \theta ^1\wedge \theta ^5-3\theta ^1\wedge \theta ^8+\tfrac{{s_5}^2(\delta M +2 L {s_5}{s_7})}{{\delta }^5}\theta ^0\wedge \theta ^2-\tfrac{{s_5}^4 L}{{\delta }^5} \theta ^0\wedge \theta ^3\nonumber \\ \mathrm{d}\theta ^2&\,=\, \theta ^1\wedge \theta ^6 - 3\theta ^2\wedge \theta ^5 - \theta ^2\wedge \theta ^8 -\tfrac{{s_5}^2(5\delta P-3 \delta M +4 L {s_5}{s_7})}{4{\delta }^5}\theta ^0\wedge \theta ^3\nonumber \\ \mathrm{d}\theta ^3&\,=\, 2\theta ^2\wedge \theta ^6 -3\theta ^3\wedge \theta ^5 + \theta ^3\wedge \theta ^8-\tfrac{{\delta }^4 U -2 {\delta }^3 R {s_5}{s_7}+{\delta }^2 Q {s_5}^2{s_7}^2+2\delta P {s_5}^3{s_7}^3 + L {s_5}^4{s_7}^4}{{\delta }^5{s_5}^5}\theta ^0\nonumber \\&\wedge \theta ^1 - \tfrac{2(\delta ^3 R-{\delta }^2 Q {s_5}{s_7} - 3 {\delta } P {s_5}^2{s_7}^2 -2 L {s_5}^3{s_7}^3)}{\delta ^5{s_5}^2}\theta ^0\wedge \theta ^2-\tfrac{\delta ^2 Q+6\delta P s_5 s_7 +6 L {s_5}^2{s_7}^2}{\delta ^3}\theta ^0\nonumber \\&\qquad \qquad \wedge \theta ^3 + \tfrac{(M-P){s_5}^2}{2{\delta }^4}\theta ^0\wedge \theta ^4\nonumber \\ \mathrm{d}\theta ^4&\,=\,6 \theta ^0\wedge \theta ^{12}+3\theta ^3\wedge \theta ^6-3\theta ^4\wedge \theta ^5+3\theta ^4\wedge \theta ^8 \end{aligned}$$

These structure equations exhibit two new relative invariant for these structures, namely *L* and $M-P$.

Let $\omega$ be any 1-adapted coframe for an integrable marked contact Engel structure, and let $\theta ^0,\theta ^1,\theta ^2,\theta ^3,\theta ^4$ be the first five forms on $\mathcal {G}^9$. Then

$$\begin{aligned} \theta ^1\wedge \theta ^2\wedge \theta ^3&=\delta ^3 {s_5}^3(\omega ^1\wedge \omega ^2\wedge \omega ^3- a \omega ^0\wedge \omega ^2\wedge \omega ^3+\tfrac{1}{2}b \omega ^0\wedge \omega ^1\wedge \omega ^3\nonumber \\&\quad - c\omega ^0\wedge \omega ^1\wedge \omega ^2). \end{aligned}$$

This shows that the kernel of $\theta ^1\wedge \theta ^2\wedge \theta ^3$ descends to a distribution $\mathcal {R}^{\sigma }=\mathrm {ker}(\phi )$ on $\mathcal {U}$, which is independent of the choice of adapted coframe, and thus invariantly associated to the marked contact twisted cubic structure. One further verifies that:

#### Proposition 4

The distribution $\mathcal {R}^{\sigma }$ is integrable if and only if $M-P=0$.

Further analysis exhibits the following tower of invariant conditions, which will be crucial for the classification of homogeneous models given in the next section, Sect. 3.4.

#### Proposition 5

Let *J*, *L*, *M*, *P*, *Q*, *R*, *S* be the functions determined by (3.2) and (3.3). The conditions

1. $$J=0$$
2. $$J=L=0$$
3. $$J=L=M=0$$
4. $$J=L=M=P=0$$
5. $$J=L=M=P=Q=0$$
6. $$J=L=M=P=Q=R=0$$
7. $$J=L=M=P=Q=R=S=0$$

are invariant under diffeomorphisms and independent of the choice of 1-adapted coframe.

### A Tree of Homogeneous Models

The remaining goal is to find all locally non-equivalent homogeneous marked contact Engel structures with symmetry group of dimension $\ge 6$. To this end, we utilize Proposition 5, which divides marked contact Engel structures into classes of mutually non-equivalent structures. Restricting to structures for which the first *i* functions from Proposition 5 vanish, the $(i+1)$-st is a relative invariant. E.g. in the branch $J=L=M=0$, the function *P* defines a relative invariant. Assuming that the invariant is non-vanishing, we then can use the *G*-action, where *G* is the structure group of the invariant coframe, to bring certain structure functions in (3.12), and further ones that arise in the reduction procedure, into normal form. The general process is referred to as *Cartan reduction*. We provide details of the Cartan reduction procedure in the case where we assume that marked Engel structure is non-integrable, i.e. $J\ne 0$, see Sect. 3.4.2 and summarize the remaining cases (see [9] for the full analysis).

#### Structures with Maximal Symmetry

Marked contact Engel structures having a symmetry algebra of maximal possible dimension are characterized by the fact that all of the structure functions $T^i{}_{jk}$ in the structure equations of the invariant coframe $(\theta ^0, \theta ^1, \theta ^2, \theta ^3, \theta ^4, \theta ^5, \theta ^6, \theta ^8, \theta ^{12})$ from Theorem 3 are constants. Then $\mathrm {d}^2\theta ^i=0$ forces them to be zero, which implies that $J=L=M=P=Q=R=S=0.$ Conversely, the condition $J=L=M=P=Q=R=S=0$ implies that all of the $T^i{}_{jk}$ vanish. In this case, the structure equations are

3.13 $$\begin{aligned} \begin{array}{llll} \mathrm{d}\theta ^0 = -6 \theta ^0\wedge \theta ^5 + \theta ^1\wedge \theta ^4 - 3\theta ^2\wedge \theta ^3 &{}\mathrm{d}\theta ^1 = - 3\theta ^1\wedge \theta ^5 - 3\theta ^1\wedge \theta ^8 \\ \mathrm{d}\theta ^2 = \theta ^1\wedge \theta ^6 - 3\theta ^2\wedge \theta ^5 - \theta ^2\wedge \theta ^8 &{} \mathrm{d}\theta ^3 =2\theta ^2\wedge \theta ^6 -3\theta ^3\wedge \theta ^5 + \theta ^3\wedge \theta ^8\\ \mathrm{d}\theta ^4=6 \theta ^0\wedge \theta ^{12}\\ \qquad \qquad +3\theta ^3\wedge \theta ^6-3\theta ^4\wedge \theta ^5+3\theta ^4\wedge \theta ^8 &{}\mathrm{d}\theta ^5= -\theta ^1\wedge \theta ^{12}\\ \mathrm{d}\theta ^6=6\theta ^2\wedge \theta ^{12}+2\theta ^6\wedge \theta ^8 &{}\mathrm{d}\theta ^8=-3\theta ^1\wedge \theta ^{12}\\ \mathrm{d}\theta ^{12}= -3\theta ^5\wedge \theta ^{12}- 3\theta ^8\wedge \theta ^{12}\,. &{} {} \end{array} \end{aligned}$$

These are the Maurer Cartan equations for the parabolic subalgebra $\mathfrak {p}_1\subset \mathfrak {g}_2$ spanned by the root vectors $E_0, E_1, E_2, E_3, E_4, E_5, E_6, E_8, E_{12}$ from the diagram in Sect. 2.3.

#### The Branch $J\ne 0$: Non-integrable Structures

We now assume that $J\ne 0$. Looking at $\mathrm{d}\theta ^1$ in Proposition 3, we see that we can normalize the coefficient $T^1{}_{24}=\tfrac{3 {s_5}^5}{\delta ^4} J$ to any non-zero value, and we shall normalize it to 3. We also see that we can normalize the coefficient $T^1{}_{02}$ to zero. This means that we restrict to a subbundle $\mathcal {G}^7\subset \mathcal {G}^9$ given by

$$\begin{aligned} s_5&=\left( \tfrac{\delta ^4}{J}\right) ^{\frac{1}{5}},\,\\&s_4=\tfrac{\delta ^4 M - 9 c \delta ^3 J s_5 s_7 - 3 \delta ^3 J_{\omega ^2} s_5 s_7 + 2 \delta ^3 L s_5 s_7 - 9 b \delta ^2 J {s_5}^2 {s_7}^2 - 9 \delta ^2 J_{\omega ^3} {s_5}^2 {s_7}^2 + 21 a \delta J {s_5}^3 {s_7}^3 - 9 \delta J_{\omega ^4} {s_5}^3 {s_7}^3 - 27 J^2 {s_5}^4 {s_7}^4}{6 \delta J {s_5}^3}. \end{aligned}$$

We pullback the forms $\theta ^0, \theta ^1, \theta ^2, \theta ^3, \theta ^4, \theta ^5, \theta ^6, \theta ^8, \theta ^{12}$ to $\mathcal {G}^7$, where they are no longer independent, and express $\theta ^8$ and $\theta ^{12}$ in terms of the remaining forms. Now we compute the structure equations of the coframe on $\mathcal {G}^7$ given by $\theta ^0,\dots ,\theta ^6$. Inspecting these structure equations shows that we can now normalize the coefficient of $\mathrm{d}\theta ^1$ at the $\theta ^1\wedge \theta ^4$ term to zero, which determines a 6-dimensional subbundle $\mathcal {G}^6\subset \mathcal {G}^7$ given by

$$\begin{aligned} s_7=\tfrac{\delta ^{\frac{1}{5}}(3 a J -J_{\omega ^4})}{14 J^{\frac{9}{5}}}. \end{aligned}$$

On this subbundle, which is parametrized by the coordinates on $\mathcal {U}$ and the fibre coordinate $\delta$, the forms $\theta ^0,\dots ,\theta ^5$ define a coframe that satisfies structure equations of the form

3.14 $$\begin{aligned} \mathrm{d}\theta ^0=&-6 \theta ^0\wedge \theta ^5+\theta ^1\wedge \theta ^4-3 \theta ^2\wedge \theta ^3\nonumber \\ \mathrm{d}\theta ^1=&\tfrac{\alpha _1}{\delta ^3} \theta ^0 \wedge \theta ^1 +\tfrac{\alpha _2}{\delta ^{\frac{12}{5}}}\theta ^0\wedge \theta ^2 + \tfrac{\alpha _3}{\delta ^{\frac{9}{5}}}\theta ^0\wedge \theta ^3 + \tfrac{\alpha _4}{\delta ^{\frac{6}{5}}} \theta ^0\wedge \theta ^4 + \tfrac{\alpha _5}{\delta ^{\frac{9}{5}}} \theta ^1\wedge \theta ^2 + \tfrac{\alpha _6}{\delta ^{\frac{6}{5}}} \theta ^1\nonumber 
\\&\wedge \theta ^3 - \tfrac{24}{5} \theta ^1\wedge \theta ^5 + 3 \theta ^2\wedge \theta ^4\nonumber \\ \mathrm{d}\theta ^2=&\tfrac{\alpha _7}{\delta ^{\frac{18}{5}}} \theta ^0 \wedge \theta ^1 + \tfrac{\alpha _8}{\delta ^3} \theta ^0 \wedge \theta ^2 + \tfrac{\alpha _9}{\delta ^{\frac{12}{5}}} \theta ^0 \wedge \theta ^3 + \tfrac{5\alpha _5}{6\delta ^{\frac{9}{5}}} \theta ^0 \wedge \theta ^4 + \tfrac{\alpha _{10}}{\delta ^{\frac{12}{5}}} \theta ^1 \wedge \theta ^2 + \tfrac{\alpha _{11}}{\delta ^{\frac{9}{5}}} \theta ^1 \nonumber \\&\wedge \theta ^3 -\tfrac{3\alpha _4 + 5 \alpha _6}{9\delta ^{\frac{6}{5}}} \theta ^1 \wedge \theta ^4 + \tfrac{\alpha _6}{3\delta ^{\frac{6}{5}}} \theta ^2 \wedge \theta ^3 - \tfrac{18}{5} \theta ^2 \wedge \theta ^5 + 2 \theta ^3 \wedge \theta ^4\nonumber \\ \mathrm{d}\theta ^3=&\tfrac{\alpha _{12}}{\delta ^{\frac{21}{5}}} \theta ^0 \wedge \theta ^1 + \tfrac{\alpha _{13}}{\delta ^{\frac{18}{5}}}\theta ^0\wedge \theta ^2+ \tfrac{\alpha _{14}}{\delta ^3} \theta ^0 \wedge \theta ^3 + \tfrac{6 \alpha _9 +75\alpha _{10}+25\alpha _2}{15 \delta ^{\frac{12}{5}}} \theta ^0 \wedge \theta ^4 +\tfrac{2(\alpha _1-3\alpha _8)}{3\delta ^3}\theta ^1\nonumber \\&\wedge \theta ^2 - \tfrac{3 \alpha _{10}+\alpha _2}{3\delta ^{\frac{12}{5}}} \theta ^1 \wedge \theta ^3 + \tfrac{\alpha _5 +6 \alpha _{11}}{3\delta ^{\frac{9}{5}}} \theta ^2 \wedge \theta ^3 - \tfrac{6 \alpha _4+10\alpha _6}{9\delta ^{\frac{6}{5}}} \theta ^2 \wedge \theta ^4 - \tfrac{12}{5} \theta ^3 \wedge \theta ^5\nonumber \\ \mathrm{d}\theta ^4=&\tfrac{\alpha _{15}}{\delta ^{\frac{24}{5}}} \theta ^0 \wedge \theta ^1 + \tfrac{\alpha _{16}}{\delta ^{\frac{21}{5}}} \theta ^0 \wedge \theta ^2 + \tfrac{\alpha _{17}}{\delta ^{\frac{18}{5}}} \theta ^0 \wedge \theta ^3 + \tfrac{\alpha _{18}}{\delta ^3} \theta ^0 \wedge \theta ^4+\tfrac{\alpha _1-3\alpha _8}{\delta ^3} \theta ^1\wedge \theta ^3 \nonumber \\&\quad -\tfrac{3\alpha _{10}+\alpha _2}{\delta ^{\frac{12}{5}}} \theta ^1 \wedge \theta ^4 \nonumber \\&\quad +\tfrac{\alpha _2}{\delta ^{\frac{12}{5}}} \theta ^2 \wedge \theta ^3 + \tfrac{\alpha _5}{\delta ^{\frac{9}{5}}} \theta ^2 \wedge \theta ^4 - \tfrac{3 \alpha _4+2\alpha _6}{3\delta ^{\frac{6}{5}}} \theta ^3 \wedge \theta ^4 - \tfrac{6}{5} \theta ^4 \wedge \theta ^5\nonumber \\ \mathrm{d}\theta ^5=&\tfrac{\alpha _{19}}{\delta ^{\frac{27}{5}}} \theta ^0 \wedge \theta ^1 + \tfrac{\alpha _{20}}{\delta ^{\frac{24}{5}}} \theta ^0 \wedge \theta ^2 + \tfrac{\alpha _{21}}{\delta ^{\frac{21}{5}}} \theta ^0 \wedge \theta ^3 + \tfrac{\alpha _{22}}{\delta ^{\frac{18}{5}}} \theta ^0 \wedge \theta ^4\nonumber \\&\quad -\tfrac{3 \alpha _{12}+\alpha _{16}}{6\delta ^{\frac{21}{5}}} \theta ^1 \wedge \theta ^2 - \tfrac{\alpha _{17}-3\alpha _7}{6 \delta ^{\frac{18}{5}}} \theta ^1 \wedge \theta ^3\nonumber \\&\quad -\tfrac{\alpha 1+\alpha _{18}}{6\delta ^3} \theta ^1 \wedge \theta ^4 + \tfrac{\alpha _8+\alpha _{14}}{2\delta ^3} \theta ^2 \wedge \theta ^3 + \tfrac{6\alpha _9+75\alpha _{10}+20\alpha _2}{30\delta ^{\frac{12}{5}}} \theta ^2 \wedge \theta ^4\nonumber \\&\quad -\tfrac{2\alpha _3 - 5 \alpha _5}{12\delta ^{\frac{9}{5}}} \theta ^3 \wedge \theta ^4, \end{aligned}$$

where $\alpha _1,\dots ,\alpha _{21}$ are the pullbacks of functions on $\mathcal {U}$, that is, as functions on $\mathcal {G}^6$ they do not depend on $\delta$.

Now we are looking for homogeneous structures with six dimensional symmetry algebra. For such structures all of the structure functions are constants. In particular, all of those that depend on $\delta$ have to be identically zero. On the other hand, one easily checks that this constant coefficient system

3.15 $$\begin{aligned} \begin{array}{ll} \mathrm{d}\theta ^0=-6 \theta ^0\wedge \theta ^5+\theta ^1\wedge \theta ^4-3 \theta ^2\wedge \theta ^3 &{} \mathrm{d}\theta ^1= - \tfrac{24}{5} \theta ^1\wedge \theta ^5 + 3 \theta ^2\wedge \theta ^4\\ \mathrm{d}\theta ^2=- \tfrac{18}{5} \theta ^2 \wedge \theta ^5 + 2 \theta ^3 \wedge \theta ^4 &{} \mathrm{d}\theta ^3= - \tfrac{12}{5} \theta ^3 \wedge \theta ^5\\ \mathrm{d}\theta ^4=- \tfrac{6}{5} \theta ^4 \wedge \theta ^5 &{} \mathrm{d}\theta ^5=0 \end{array} \end{aligned}$$

is closed, that is, $\mathrm{d}^2\theta ^i=0$, for all $i=0,1,2,3,4,5$. This means that there is a unique local model with 6-dimensional symmetry algebra in this branch.

There may be homogeneous models with 5-dimensional symmetry algebra in this branch as well.

#### The Branch $J=0$, $L\ne 0$

There is a locally unique homogeneous model in this branch. It has a 5-dimensional symmetry algebra with Maurer–Cartan equations of the form

3.16 $$\begin{aligned} \begin{aligned} \mathrm{d}\theta ^0&= -\tfrac{5}{6} \theta ^0\wedge \theta ^3 -24 \theta ^0\wedge \theta ^4\\&\quad \quad + \theta ^1\wedge \theta ^4 -3 \theta ^2\wedge \theta ^3 \mathrm{d}\theta ^1\qquad = \theta ^0\wedge \theta ^3 -\tfrac{2}{3}\theta ^1\wedge \theta ^3 -30 \theta ^1\wedge \theta ^4\\ \mathrm{d}\theta ^2&= -\tfrac{1}{2} \theta ^2\wedge \theta ^3 -18 \theta ^2\wedge \theta ^4 \mathrm{d}\theta ^3 = -6\theta ^3\wedge \theta ^4 \\ \mathrm{d}\theta ^4&=\tfrac{1}{6} \theta ^3\wedge \theta ^4. \\ \end{aligned} \end{aligned}$$

#### The Branch $J=L=0$, $M\ne 0$, $P\ne 0$

There are exactly two locally non-equivalent homogeneous models in this branch. They have 5-dimensional symmetry algebras with Maurer–Cartan equations ($\epsilon =\pm 1$)

3.17 $$\begin{aligned}&\mathrm{d}\theta ^0 = -\tfrac{15}{2} \theta ^0\wedge \theta ^2 -\tfrac{1}{6}\epsilon \theta ^0\wedge \theta ^4 + \theta ^1\wedge \theta ^4 -3 \theta ^2\wedge \theta ^3\nonumber \\&\mathrm{d}\theta ^1 = \epsilon \theta ^0\wedge \theta ^2 -3 \theta ^1\wedge \theta ^2 -\tfrac{1}{3}\epsilon \theta ^1\wedge \theta ^4\nonumber \\&\mathrm{d}\theta ^2 = \tfrac{1}{4} \theta ^0\wedge \theta ^1 -\tfrac{1}{12}\epsilon \theta ^0\wedge \theta ^3-\tfrac{1}{2}\theta ^1\wedge \theta ^3-\tfrac{1}{6}\epsilon \theta ^2\wedge \theta ^4 \nonumber \\&\mathrm{d}\theta ^3 = \tfrac{9}{2}\theta ^0\wedge \theta ^2+\tfrac{1}{6}\epsilon \theta ^0\wedge \theta ^4+9\epsilon \theta ^1\wedge \theta ^2+3\theta ^2\wedge \theta ^3 \nonumber \\&\mathrm{d}\theta ^4=-\tfrac{27}{4}\epsilon \theta ^0\wedge \theta ^1+\tfrac{9}{4}\theta ^0\wedge \theta ^3+\tfrac{27}{2}\epsilon \theta ^1\wedge \theta ^3+\tfrac{9}{2}\theta ^2\wedge \theta ^4 . \end{aligned}$$

#### The Branch $J=L=0$, $M\ne 0$, $P=0$

There are exactly two locally non-equivalent homogeneous structures in this branch, which have structure equations

3.18 $$\begin{aligned} \begin{array}{ll} \mathrm{d}\theta ^0 =-6\theta ^0\wedge \theta ^5+\theta ^1\wedge \theta ^4-3\theta ^2\wedge \theta ^3 &{}\mathrm{d}\theta ^1 = \epsilon \theta ^0\wedge \theta ^2-12\theta ^1\wedge \theta ^5 \\ \mathrm{d}\theta ^2 =\tfrac{3}{4} \epsilon \theta ^0\wedge \theta ^3+\theta ^1\wedge \theta ^6-6\theta ^2\wedge \theta ^5 &{}\mathrm{d}\theta ^3 =\tfrac{1}{2}\epsilon \theta ^0\wedge \theta ^4+2\theta ^2\wedge \theta ^6 \\ \mathrm{d}\theta ^4=6\theta ^0\wedge \theta ^{12}+3\theta ^3\wedge \theta ^6+6\theta ^4\wedge \theta ^5 &{}\mathrm{d}\theta ^5=-\tfrac{1}{12}\epsilon \theta ^0\wedge \theta ^6-\theta ^1\wedge \theta ^{12}+\tfrac{1}{12}\epsilon \theta ^2\wedge \theta ^4\\ \mathrm{d}\theta ^6=6\theta ^2\wedge \theta ^{12}-\tfrac{3}{4}\epsilon \theta ^3\wedge \theta ^4-6\theta ^5\wedge \theta ^6 &{}\mathrm{d}\theta ^{12}=\tfrac{1}{6}\epsilon \theta ^4\wedge \theta ^6-12\theta ^5\wedge \theta ^{12}. \\ \end{array}\nonumber \\ \end{aligned}$$

These are Maurer–Cartan equations for $\mathfrak {sl}(3,\mathbb {R})$ if $\epsilon <0$ and Maurer–Cartan equations for $\mathfrak {su}(2,1)$ if $\epsilon > 0$.

#### The Branch $J=L=M=0$, $P\ne 0$

There are no homogeneous models with symmetry algebra of dimension $\ge 6$ in this branch. There may be homogeneous models with 5-dimensional symmetry algebra.

#### The Branch $J=0$, $L=0$, $M=0$, $P=0$, $Q\ne 0$

There is a locally unique homogeneous model with symmetry algebra of dimension $\ge 6$ in this branch. The structure equations of the model

3.19 $$\begin{aligned} \begin{array}{ll} \mathrm{d}\theta ^0 = \theta ^1\wedge \theta ^4 -3 \theta ^2\wedge \theta ^3 &{}\mathrm{d}\theta ^1 = \tfrac{1}{2}\theta ^0\wedge \theta ^1 -3 \theta ^1\wedge \theta ^8\\ \mathrm{d}\theta ^2 = \tfrac{1}{2}\theta ^0\wedge \theta ^2 - \theta ^2\wedge \theta ^8 &{}\mathrm{d}\theta ^3 = -\tfrac{1}{2}\theta ^0\wedge \theta ^3 + \theta ^3\wedge \theta ^8\\ \mathrm{d}\theta ^4= -\tfrac{1}{2}\theta ^0\wedge \theta ^4 +3 \theta ^4\wedge \theta ^8 &{}\mathrm{d}\theta ^8= -\tfrac{1}{2}\theta ^1\wedge \theta ^4 +\tfrac{1}{2} \theta ^2\wedge \theta ^3 \end{array} \end{aligned}$$

are the Maurer–Cartan equations for $\mathfrak {sl}(2,\mathbb {R})\oplus \mathfrak {sl}(2,\mathbb {R})$ with respect to a basis of left-invariant forms. There may be homogeneous models with 5-dimensional symmetry algebras in this branch as well.

### Summary

We summarize the main results of this section in the following theorem (see also Table 1).

#### Theorem 2

1. Up to local equivalence, there exists a unique maximally symmetric marked contact Engel structure. Its infinitesimal symmetry algebra is isomorphic to the 9-dimensional parabolic subalgebra $\mathfrak {p}_1$ of $\mathfrak {g}_2$. The maximally symmetric structure is characterized by
  $$\begin{aligned} J=L=M=P=Q=R=S=0. \end{aligned}$$
2. Up to local equivalence, there are precisely two homogeneous marked contact Engel structures with 8-dimensional infinitesimal symmetry algebra. The infinitesimal symmetry algebras are isomorphic to $\mathfrak {sl}(3,\mathbb {R})$ and $\mathfrak {su}(1,2)$, respectively. The structures are characterized by
  $$\begin{aligned} J=L=P=Q=0\quad \hbox {and}\quad M\ne 0. \end{aligned}$$
3. There are no homogeneous marked contact Engel structures with 7-dimensional infinitesimal symmetry algebra.
4. Up to local equivalence, there are precisely two homogeneous marked contact Engel structures with 6-dimensional infinitesimal symmetry algebras. The respective Maurer–Cartan equations are given in (3.15) and (3.19); the second symmetry algebra is isomorphic to $\mathfrak {sl}(2,\mathbb {R})\oplus \mathfrak {sl}(2,\mathbb {R})$.
5. There are examples of homogeneous marked contact Engel structures with 5-dimensional infinitesimal symmetry algebra, whose Maurer–Cartan equations are given in (3.16) and (3.17).

**Table 1:**
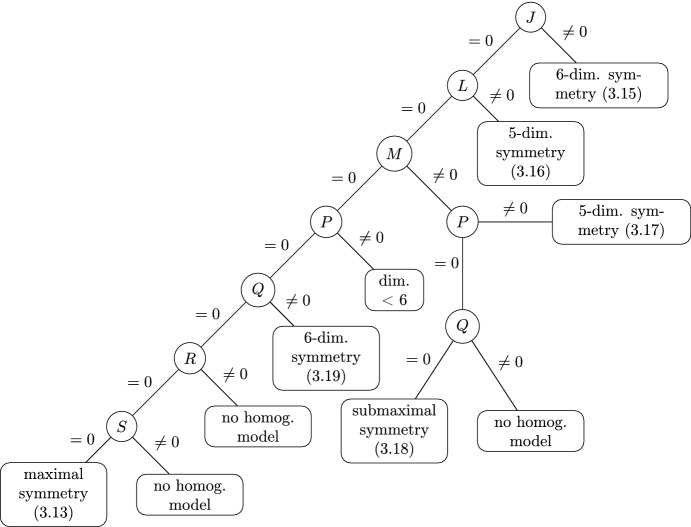
The following graph shows the maximal symmetry dimension for homogeneous models in various branches of marked contact Engel structures

## Generalizations

It is clear that the concept of a marked contact Engel structure can be generalized in several directions. One natural generalization is to replace the contact Engel structure by a general (curved) contact twisted cubic structure. Such a generalization is analogous to considering null-congruence structures in a general 4-dimensional spacetime, rather than restricting to conformally flat ones.

### Definition 6

A *marked contact twisted cubic structure* $(\mathcal {M},\mathcal {C},{\varvec{\upgamma }}, \sigma )$ is a contact twisted cubic structure $(\mathcal {M},\mathcal {C},{\varvec{\upgamma }})$ together with a section $\sigma :\mathcal {M}\rightarrow {\varvec{\upgamma }}\subset \mathbb {P}(\mathcal {C})$ of the bundle $\,\mathbb {R}\mathbb {P}^1\rightarrow {\varvec{\upgamma }}\rightarrow \mathcal {M}$ of twisted cubics.

The detailed study of these structures becomes more involved, but some basic results can be easily derived using some Tanaka theory [10, 18, 19] and algebraic facts that we briefly outline below. In particular, it follows from these considerations that the marked contact Engel structure with $\mathfrak {p}_1$-symmetry is a maximally symmetric model in the class of *all* marked contact twisted cubic structures.

The Lie algebra $\mathfrak {g}$ of $\mathrm {G}_2$ admits a unique contact grading

4.1 $$\begin{aligned} \mathfrak {g}=\mathfrak {g}_{-2}\oplus \mathfrak {g}_{-1}\oplus \mathfrak {g}_0\oplus \mathfrak {g}_{1}\oplus \mathfrak {g}_2, \end{aligned}$$

where $\mathfrak {m}=\mathfrak {g}_{-2}\oplus \mathfrak {g}_{-1}$ is a 5-dimensional Heisenberg Lie algebra (see [6, 19] and the root diagram in Sect. 2.3). We denote by $G_0\subset \mathrm {G}_2$ the subgroup that preserves the grading via the adjoint representation; it is isomorphic to $\mathrm {GL}(2,\mathbb {R})$ and its Lie algebra is the grading component $\mathfrak {g}_0$. As a representation of the semisimple part of $G_0$, we have $\mathfrak {g}_{-1}\cong \bigodot ^3\mathbb {R}^2$. In particular, the $G_0$ action on $\mathfrak {g}_{-1}$ preserves a unique twisted cubic cone $\hat{\upgamma }$ and a unique conformal symplectic structure $[\omega ]$; the latter coincides with the one corresponding to the component of the Lie bracket $[,]:\Lambda ^2\mathfrak {g}_{-1}\rightarrow \mathfrak {g}_{-2}\cong \mathbb {R}$. We denote by

4.2 $$\begin{aligned} B\subset \mathrm {GL}(2,\mathbb {R})\cong G_0\subset \mathrm {CSp}(\mathfrak {g}_{-1}) \end{aligned}$$

the subgroup preserving a line $\ell \subset \hat{\upgamma }\subset \mathfrak {g}_{-1}$.

A marked contact twisted cubic structure can be equivalently described as a contact structure $\mathcal {C}\subset T{\mathcal M}$ on a 5-manifold together with a reduction of structure group of the graded frame bundle of the contact structure with respect to the inclusion (4.2). In the terminology of [10], it is a filtered *G*-structures of type $\mathfrak {m}$, where *G* is the subgroup $B\subset \mathrm {CSp}(\mathfrak {g}_{-1})$ of (4.2) and $\mathfrak {m}=\mathfrak {g}_{-2}\oplus \mathfrak {g}_{-1}$ the 5-dimensional Heisenberg algebra.

Tanaka’s general theory tells us that given any subalgebra $\mathfrak {q}_0\subset \mathfrak {csp}(\mathfrak {g}_{-1})$, there exists graded Lie algebra $\mathfrak {g}(\mathfrak {m},\mathfrak {q}_0)=\bigoplus _i\mathfrak {g}(\mathfrak {m}, \mathfrak {q}_0)_i$, called the (algebraic) *Tanaka prolongation* of the pair $(\mathfrak {m},\mathfrak {q}_0)$, uniquely determined by the following conditions:

1. The non-positive part of $\mathfrak {g}(\mathfrak {m},\mathfrak {q}_0)$ is $\mathfrak {m}\oplus \mathfrak {q}_0$.
2. If $X\in \mathfrak {g}(\mathfrak {m},\mathfrak {q}_0)_i$ for some $i>0$ satisfies $[X,\mathfrak {m}_{-1}]=\{0\}$, then $X=0$.
3. $\mathfrak {g}(\mathfrak {m},\mathfrak {q}_0)$ is maximal among the graded Lie algebras satisfying (1) and (2).

It is well known, see [19], that the Tanaka prolongation of the pair $(\mathfrak {m},\mathfrak {g}_0)$, where $\mathfrak {g}_0\subset \mathfrak {csp}(\mathfrak {g}_{-1})$ is the zero graded component in (4.1), recovers the exceptional Lie algebra $\mathfrak {g}$ of $\mathrm {G}_2$ with its contact grading (4.1). For a subalgebra $\mathfrak {q}_0\subset \mathfrak {g}_0$, the Tanaka prolongation $\mathfrak {q}=\mathfrak {g}(\mathfrak {m},\mathfrak {q}_0)$ of the pair $(\mathfrak {m},\mathfrak {q}_0)$ can be identified with a graded subalgebra of $\mathfrak {g}=\mathfrak {g}(\mathfrak {m},\mathfrak {g}_0)$, where $\mathfrak {q}_i=\mathfrak {g}_i$ for $i\le 0$ and $\mathfrak {q}_{i}=\{X\in \mathfrak {g}_{i}:\, [X,\mathfrak {g}_{-1}]\subset \mathfrak {q}_{i-1} \}$ for $i>0$.

### Proposition 6

Let $\mathfrak {g}=\mathfrak {g}_{-2}\oplus \mathfrak {g}_{-1}\oplus \mathfrak {g}_0\oplus \mathfrak {g}_{1}\oplus \mathfrak {g}_2$ be the Lie algebra of $\mathrm {G}_2$ equipped with its contact grading, $\mathfrak {m}=\mathfrak {g}_{-2}\oplus \mathfrak {g}_{-1}$ the 5-dimensional Heisenberg Lie algebra, and let $\mathfrak {q}_0\subset \mathfrak {g}_0\cong \mathfrak {gl}(2,\mathbb {R})$ be the subalgebra preserving a highest weight line $\ell \subset \hat{\upgamma }\subset \mathfrak {g}_{-1}$. Then the Tanaka prolongation $\mathfrak {q}$ of $(\mathfrak {m},\mathfrak {q}_0)$ is isomorphic to the 9-dimensional parabolic subalgebra $\mathfrak {p}_1\subset \mathfrak {g}$ (with the grading depicted below).

### Proof

Consider the root diagram of $\mathrm {G}_2$. 
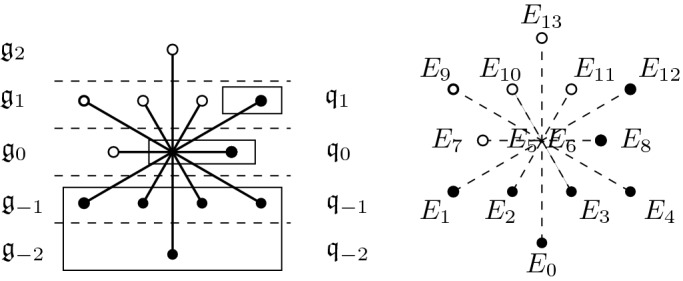


Let $\mathfrak {q}=\mathfrak {q}_{-2}\oplus \mathfrak {q}_{-1}\oplus \mathfrak {q}_0\oplus \mathfrak {q}_1$ be the subalgebra of $\mathfrak {g}$ spanned by the Cartan subalgebra and all root spaces corresponding to black nodes. Then $\mathfrak {q}$ is a graded Lie algebra satisfying properties (1) and (2) characterizing the Tanaka prolongation. Moreover, there is no proper subalgebra $\mathfrak {q}'\subset \mathfrak {g}$ containing $\mathfrak {q}$. This can be deduced from the above root diagram, by observing that any subalgebra $\mathfrak {q}'$ containing $\mathfrak {q}$ and in addition a root space corresponding to a white root has to be all of $\mathfrak {g}$. Alternatively, it follows from the fact that a Lie algebra of root type $\mathrm {G}_2$ has no subalgebra of dimension bigger than 9. Hence property (3) is satisfied as well. $\square$

Having established Proposition 6 and a description of marked contact twisted cubic structures in the setting of filtered *G*-structures, Tanaka theory then provides a procedure to associate to any marked contact twisted cubic structure $(\mathcal {M},\mathcal {C},{\varvec{\upgamma }},\sigma )$, in a natural manner, a 9-dimensional bundle $\mathcal {G}\rightarrow {\mathcal M}$ together with a coframe $\omega$ on $\mathcal {G}$. This in particular implies the following:

### Corollary 2

The infinitesimal symmetries of a marked contact twisted cubic structure form a Lie algebra of dimension $\le \mathrm {dim}(\mathfrak {p}_1)=9$.

## Footnotes

1 Contact Engel structures should not be confused with Engel distributions, sometimes also called Engel structures, which are maximally non-integrable rank 2 distributions on 4-dimensional manifolds.

2 The analogy is even more striking if one realizes that a contact Engel structures can be equivalently defined by the *conformal class* of the fourth rank tensor $\Upsilon =3(\alpha ^2)2(\alpha ^3)^2-4\alpha ^1(\alpha ^3)^3-4(\alpha ^2)^3\alpha ^4+6\alpha ^1\alpha ^2\alpha ^3\alpha ^4-(\alpha ^1)^2(\alpha ^4)^2$ on $\mathcal {C}$. The null directions defined here are special null directions for this tensor.

3 The choice of sign of the function *t* we use to parameterize marked Engel structures comes from the fact that for this paper we primarily care about the coframe (2.11) adapted to the structure.

4 We state our theorem in parallel to Penrose’s formulation of the original Kerr theorem, as in [14][Theorem 7.4.8].

5 For the reason behind the seemingly unreasonable numbering of the 1-forms $\theta ^i$ see Sect. 3.4.1 about the maximally symmetric model and the root diagram from Sect. 2.3.
